# Genome-Wide Association Study Pinpoints Novel Candidate Genes Associated with the Gestation Length of the First Parity in French Large White Sows

**DOI:** 10.3390/ani15030447

**Published:** 2025-02-06

**Authors:** Dongdong Duan, Shenping Zhou, Zhenyu Wang, Chuanmin Qiao, Jinyi Han, Mengyu Li, Hao Zhou, Xinjian Li, Wenshui Xin

**Affiliations:** 1 Sanya Institute, Hainan Academy of Agricultural Sciences, Sanya 572025, China; duandongd@126.com (D.D.); 18819427274@163.com (S.Z.); wzyhan2017@163.com (Z.W.); qiaochuanmin@hnaas.org.cn (C.Q.); hjy7jane@126.com (J.H.); mengyuli2020@163.com (M.L.); 13703822441@163.com (H.Z.); 2 Institute of Animal Science and Veterinary Medicine, Hainan Academy of Agricultural Sciences, Haikou 571100, China

**Keywords:** gestation length, novel QTLs, novel genes, French Large White sows

## Abstract

Gestation length (GL) is a critical indicator of reproductive performance in sows and is closely associated with other reproductive traits, such as total number born (TNB) and number born alive (NBA). Despite its importance, the genetic mechanisms underlying GL and its impact on reproductive traits remain poorly understood. In this study, we investigated the relationship between GL and reproductive traits using 7013 farrowing records and conducted an imputed whole-genome sequence-based genome-wide association study (GWAS) for GL in first-parity sows, involving 3005 French Large White sows. Our findings revealed that the heritability of GL ranged from 0.22 to 0.26. Both excessively short and long GLs were associated with negative impacts on TNB, NBA, and other reproductive traits. A total of 64 SNPs exceeded the significance threshold, leading to the identification of two novel quantitative trait loci (QTLs) on chromosome 5 (QTL-1: 15.29–15.39 Mb and QTL-2: 12.86–12.94 Mb) and three promising candidate genes: *TROAP*, *RFX4*, and *ADCY6*. Gene ontology and KEGG pathway enrichment analyses revealed that these candidate genes are enriched in key biological processes, including ovarian steroidogenesis, the GnRH signaling pathway, and the regulation of cAMP biosynthesis, all of which are crucial for gestation and pregnancy maintenance. These findings improve our understanding of the genetic architecture of GL in sows and offer valuable genetic markers for enhancing reproductive efficiency in breeding programs.

## Simple Summary

In modern pig production systems, the reproductive performance of sows significantly influences the economic efficiency of practical pig farming. Gestation length, a heritable reproductive trait, is closely linked to sow productivity, highlighting the importance of identifying single nucleotide polymorphisms (SNPs) and genes associated with this trait. In this study, we performed a genome-wide association analysis (GWAS) using imputed whole-genome sequence data to investigate gestation length in a large cohort of French Large White sows. The heritability of gestation length was estimated to be within the range of 0.22 and 0.26. Our analysis identified two novel quantitative trait loci (QTLs) located on chromosome 5 (QTL-1: 15.29–15.39 Mb and QTL-2: 12.86–12.94 Mb) and three promising candidate genes: *TROAP*, *RFX4*, and *ADCY6*. These findings enhance our understanding of the genetic architecture underlying gestation length in pigs and may provide valuable insights for improving reproductive performance through genomic selection.

## 1. Introduction

The reproductive performance of sows directly impacts pork production efficiency and dictates the economic profitability of breeding enterprises. As such, the genetic improvement of reproductive traits has long been a central focus of pig breeding programs. Gestation length (GL), which is the number of days from the last artificial insemination to farrowing, typically averages 114 days (commonly referred to as 3 months, 3 weeks, and 3 days) [1]. It is a pivotal trait influencing reproductive efficiency, as it plays a crucial role in the development and maturation of the piglets’ organs. Abnormal gestation lengths, whether excessively short or prolonged, are often associated with compromised sow fertility and impaired piglet performance. Therefore, understanding the genetic mechanisms regulating GL is essential for mitigating such abnormalities and enhancing overall reproductive success.

GL is a complex quantitative trait with moderate heritability (*h*^2^ ≈ 0.39) and is influenced by both genetic and environmental factors [2,3]. Traditional breeding methods aimed at improving GL are often time-consuming and labor-intensive. In contrast, genetic and molecular breeding approaches hold promise as more efficient and effective strategies for optimizing GL in sows [4]. According to the Pig QTLdb (https://www.animalgenome.org (accessed on 23 June 2024)) [5], 286 quantitative trait loci (QTLs) have been reported to be associated with GL, accounting for only 4.2% of the total QTLs linked to reproductive traits. Despite these findings, the genetic mechanisms underlying GL remain poorly understood, and many potential genomic variants associated with GL are yet to be identified.

Genome-wide association studies (GWAS) provide a powerful statistical framework for associating single nucleotide polymorphism (SNP) markers across the genome with complex traits, thereby identifying genetic variants most likely to influence the phenotypes of interest [6,7]. In pigs, GWAS are essential for understanding the genetic basis of important economic traits [8,9,10,11,12]. The effectiveness of GWAS increases with larger sample sizes and higher SNP densities; however, high-coverage whole-genome sequencing for thousands of animals remains prohibitively expensive. Genotype imputation offers a cost-effective alternative, enabling low-density SNP genotype data to be imputed to whole-genome sequence resolution using a reference panel [13,14]. This approach enhances the resolution of GWAS, facilitating the identification of causal variants by narrowing the genomic regions associated with target traits.

In this study, we utilized first-parity farrowing data from 7013 sows and performed genotyping on 3005 French Large White sows with valid GL records and ear tissue samples. Our objectives were threefold: (I) To elucidate the relationship between GL and reproductive traits in sows; (II) To identify SNPs and QTLs associated with GL; (III) To annotate and identify potential candidate genes underlying GL. The results of this study provide a theoretical foundation for the molecular breeding of pigs, with the ultimate goal of improving GL and enhancing reproductive efficiency.

## 2. Materials and Methods

### 2.1. Animals and Phenotypes

Detailed farrowing data from the first parity of 7013 French Large White sows were collected, including gestation length (GL), total number born (TNB), number born alive (NBA), number of healthy piglets (NHB), average birth weight of piglets (ABW), and litter birth weight (LBW). The complete pedigree data were obtained from the nucleus breeding farm of XinDa Co., Ltd., Zhengzhou, China, spanning the years 2018 to 2023. Ear tissue samples were collected from 3005 sows for genetic analysis. All pigs were raised under standardized conditions. Gestation length was defined as the time (in days) from the last insemination to natural farrowing for the current parity. All phenotypic traits were included in subsequent analyses.

### 2.2. Genotyping and Quality Control

Genomic DNA was extracted from the ear tissue of 3005 Large White sows using a commercial DNA extraction kit. The quality of the DNA was assessed through electrophoresis and light absorption ratio measurements (A260/280). DNA samples were diluted to a final concentration of 50 ng/μL and genotyped using the Porcine 50K SNP Bead Chip (COMPASS BIOTECHNOLOGY, Beijing, China). A total of 51,315 SNPs were initially obtained. Quality control of the raw genotype data was performed using PLINK software (v1.90) [15] under the following criteria: individual call rates > 95%, SNP call rates > 90%, minor allele frequency (MAF) > 1%, and Hardy–Weinberg equilibrium (HWE) *p*-value > 1.00 × 10^−6^ [16,17]. After filtering and removal of unmapped SNPs, 37,006 SNPs and 3005 individuals were retained for further analysis.

### 2.3. Genotype Imputation and PCA Analysis

Whole-genome phasing and imputation were performed using Beagle software (v5.2) [18], with default parameters except for adjustments to the effective population size (ne = 210), window size (80 cM), and overlap size (70 cM). The squared DR^2^ value was used to assess imputation accuracy. Public genomic data were used as the reference population [19]. After imputation and subsequent quality control (MAF > 0.01), 13,099,033 SNPs were retained for downstream analyses. Principal component analysis (PCA) was conducted using PLINK software [15].

### 2.4. Statistics of Phenotype and Genetic Parameters Estimation

Descriptive statistics (e.g., mean, maximum, and minimum values) were calculated, and Pearson’s correlation coefficient (R) along with corresponding *p*-values were used to evaluate relationships between GL and other reproductive traits using R software (v4.4.1). Genetic and residual variances were estimated using BLUPF90+ software (v1.70) with a single-trait animal model [20]:

$$y_{i}={ysb}_{i}+u_{i}+e_{i}$$

where $y_{i}$ represents the phenotypic values of gestation length for the *i*-th animal; ${ysb}_{i}$ is the vector of fixed effects of *i*-th animal (including year, season, and farrowing batch); $u_{i}$ is the additive random effect of the *i*-th animal; and $e_{i}$ is the residual effect.

Heritability was calculated using the following formula:

$$h^{2}=\frac{V_{g}}{V_{g}+V_{e}}$$

where $V_{g}$ is the genetic variance, and $V_{e}$ is the residual variance.

### 2.5. Genome-Wide Association Analysis

Genome-wide association analysis (GWAS) for gestation length was conducted using the mixed linear model implemented in GEMMA (v0.98) software [21]. The statistical model applied was as follows:

y=Wα+Xβ+u+ε

where y is the vector of phenotypic values for all individuals; W is the covariate matrix; α is the vector of corresponding coefficients, including the intercept; X is the vector of marker genotypes; β represents the effect size of the marker; u an *m* × 1 vector of random effects $u\sim {MVN}_{n}(0,\lambda \tau^{-1}K)$; and ε is the vector of random residuals ($\varepsilon \sim {MVN}_{n}(0,\tau^{-1}ln)$). Here, λ is the ratio of variance components; $\tau^{-1}$ is the variance of the residual errors; K is the known *n × n* relatedness matrix calculated in previous step; and I is the identity matrix. ${MVN}_{n}$ denotes the n-dimensional multivariate normal distribution.

Farrowing year, season, and batch were used as fixed effects. Genome-wide significance thresholds were set at 5.0 × 10^−8^, and suggestive significance thresholds at 1.0 × 10^−6^, as described by Yan and Wang [22,23]. The inflation factor was computed by using the R package, and Manhattan and Q–Q plots were generated with the rMVP package to assess false-positive rates [24]. The QTL was represented by the logarithm of odds (LOD) score, which represents the disparity of likelihoods between the alternative hypothesis (presence of a QTL) and null hypothesis (absence of a QTL), calculated as −log_10_(*p*-value) [25].

The proportion of variance explained (PVE) by top SNP was calculated as follows:

$$PVE=\frac{2\beta^{2}MAF(1-MAF)}{2\beta^{2}MAF(1-MAF)+{\left(se(\beta )\right)}^{2}2NMAF(1-MAF)}$$

where β is the effect of markers from GWAS, MAF is the minor allele frequency, and se(β) is the standard error of effect of variants, and N is the sample size.

### 2.6. Linkage Disequilibrium Analysis

Linkage disequilibrium (LD) around top SNPs was analyzed, and LD blocks were constructed using LDBlockShow (v1.40) software [26]. Pairwise LD for all variants in the chromosomal region was estimated using PLINK (v1.90) [15].

### 2.7. Variants Annotation and Gene Enrichment Analysis

Significant SNPs were annotated using the Ensembl database (http://ensembl.org/Sus_scrofa/Info/Index, accessed on 5 October 2024) and the Ensembl Variant Effect Predictor (https://asia.ensembl.org/info/docs/tools/vep/index.html, VEP, accessed on 5 October 2024) [27]; Annotated genes were identified as candidate genes. To investigate the biological roles of these genes, we conducted Gene Ontology (GO) and Kyoto Encyclopedia of Genes and Genomes (KEGG) pathway analyses using the KOBAS platform (http://bioinfo.org/kobas, accessed on 5 October 2024) [28]. Functions of known candidate genes in significant regions were further validated through literature review.

## 3. Results

### 3.1. Descriptive Statistics of Phenotypic Data

**Figure 1:**
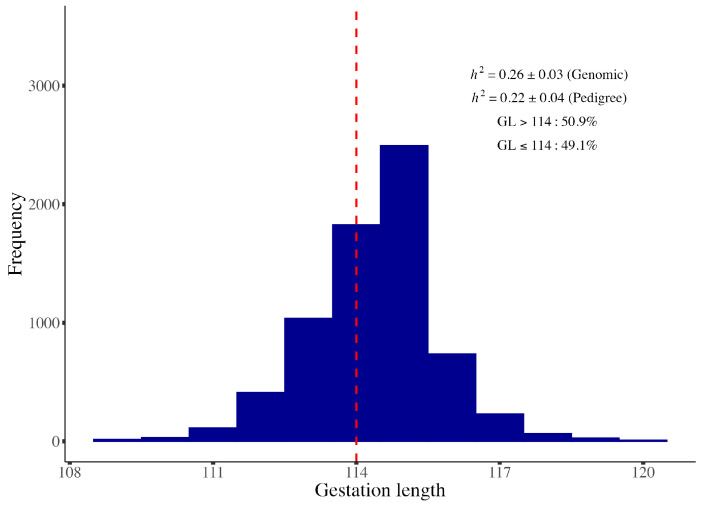
GL phenotype distribution and descriptive statistics.

The distribution and descriptive statistics of GL phenotypes are presented in Figure 1. The average GL in this population was 114.23 days, with a range from 109 to 120 days. The estimated heritability of GL was 0.26 ± 0.03 (genomic-based) and 0.22 ± 0.04 (pedigree-based). Significant correlations were observed between GL and other reproductive traits. For sows with GL ≤ 114 days, the GL was positively correlated with NBA (*cor* = 0.114, *p*-value = 8.5 × 10^−30^), NHB (*cor* = 0.128, *p*-value = 2.03 × 10^−37^), and LWB (*cor* = 0.08, *p*-value = 5.08 × 10^−15^). Conversely, for GL ≥ 114 days, significant negative correlations were found with NBA (*cor* = −0.127, *p*-value = 3.13 × 10^−72^), TNB (*cor* = −0.116, *p*-value = 2.05 × 10^−60^), NHB (*cor* = −0.099, *p*-value = 1.13 × 10^−44^), and LWB (*cor* = −0.048, *p*-value = 2.94 × 10^−11^). Additionally, the GL was negatively correlated with SN (*cor* = −0.201, *p*-value = 5.56 × 10^−91^) for GL ≤ 114 days, while positive correlations were observed with SN (*cor* = 0.042, *p*-value = 2.09 × 10^−9^) and ABW (*cor* = 0.09, *p*-value = 6.54 × 10^−36^) for GL ≥ 114 days. No significant correlations were detected between GL and TNB (*cor* = 0.01, *p*-value = 0.299) or ABW (*cor* = 0.014, *p*-value = 0.178) for GL ≤ 114 days (Table 1, Figure S1). These findings indicate that both excessively short and excessively long gestation lengths negatively impact the reproductive performance in sows.

### 3.2. GWAS and Fine-Mapping of QTLs

**Figure 2:**
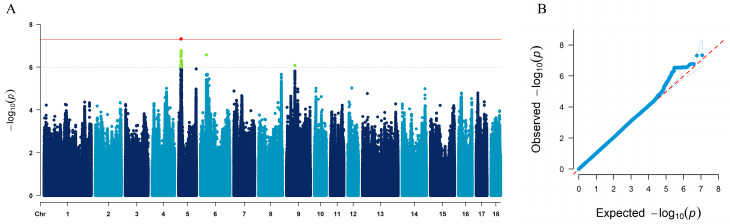
Manhattan and Q–Q plots for GWAS of gestation length (GL) in French Large White pigs. (**A**) The Manhattan plot shows significant SNPs associated with GL. The solid red line indicates the genome-wide significance threshold (*p*-value = 5.00 × 10^−8^), and the dashed line indicates the suggestive significance threshold (*p*-value = 1.00 × 10^−6^). (**B**) The Q–Q plot demonstrates minimal population stratification with a genomic inflation factor of 1.051.

**Figure 3:**
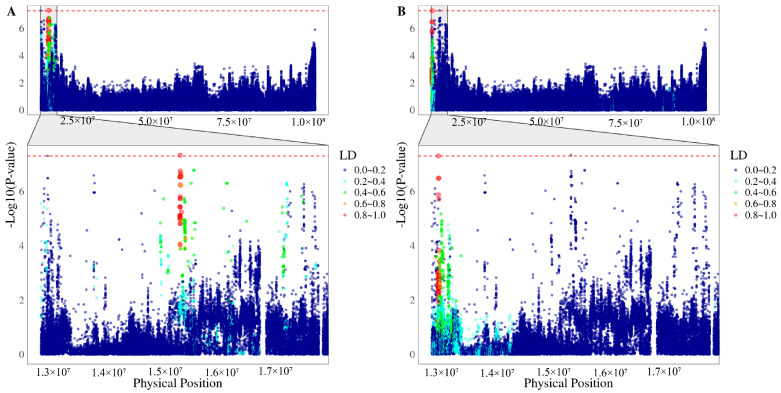
Regional association plots and linkage disequilibrium around the two leading SNPs. (**A**) LD plot for 5_15295033 (*p*-value = 4.69 × 10^−8^), defining QTL-1: 15290495–15392868 bp; (**B**) LD plot for 5_12923575 (*p*-value = 4.92 × 10^−8^), defining QTL-2: 12868808–12949729 bp. The red dashed line indicates genome-wide significance threshold (5 × 10^−8^).

The average imputation accuracy across the whole genome was 0.78, and PCA indicated no evidence of population stratification (Figure S3). A total of 64 SNPs were significantly associated with gestation length (Figure 2A, Table 2). Of these, 62 SNPs surpassed the suggestive significance threshold (*p*-value < 1 × 10^−6^), and 2 SNPs exceeded the genome-wide significance threshold (*p*-value < 5 × 10^−8^). The top SNP, 5_15295033 (*p*-value = 4.69 × 10^−8^, *PVE* = 0.92%), is located on SSC5. Among the 64 significant SNPs, 61 were mapped to SSC5, 1 SNP to SSC6 (6_34420154, *PVE* = 0.83%), and 2 SNPs to SSC9 (9_48574739 and 9_48574749, both with *PVE* = 0.75%). The quantile–quantile (Q–Q) plot showed that the observed *p*-values were well-aligned with the expected distribution, with a genomic inflation factor of 1.051, suggesting minimal confounding due to population stratification or cryptic relatedness (Figure 2B). Using a 2-LOD drop method, a 4.9 Mb genomic region on SSC5 was identified around the top SNPs, containing two independent QTLs surpassing the genome-wide significance threshold (−log_10_5 × 10^−8^) (Figure 3A,B).

QTL-1: 15290495–15392868 bp, centered around 5_15295033 (*p*-value = 4.69 × 10^−8^).

QTL-2: 12868808–12949729 bp, centered around 5_12923575 (*p*-value = 4.92 × 10^−8^).

**Figure 4:**
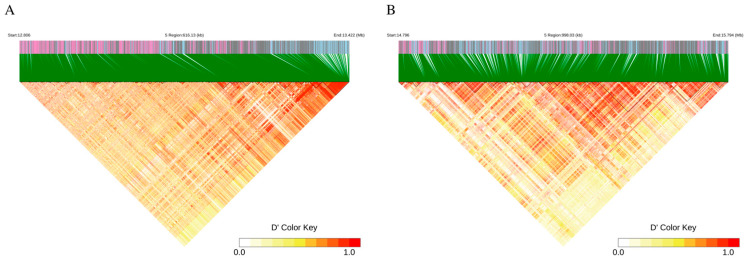
Linkage disequilibrium (LD) blocks within the 15.29–15.54 Mb region on chromosome 5. (**A**) LD blocks surrounding chr5_12923575. (**B**) LD blocks surrounding chr5_15295033.

Linkage disequilibrium (LD) analysis using LDBlockShow revealed 548 LD blocks within a ±500 Kb region of the two leading SNPs on SSC5 (Figure 4A, chr5_12923575: *p*-value = 4.92 × 10^−8^; Figure 4B, chr5_15295033: *p*-value = 4.69 × 10^−8^). The LD block lengths ranged from 0.011 Kb to 150.45 Kb. The differences in genotypes of the top SNPs on SSC5 caused significant variations in gestation length (Figure S2).

### 3.3. Candidate Gene Search and Enrichment Analysis

**Figure 5:**
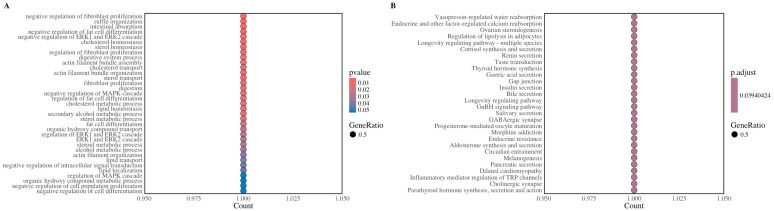
Enrichment analysis of candidate genes. (**A**) Significant Gene Ontology (GO) terms significantly enriched for candidate genes associated with GL; (**B**) Significant KEGG pathways significantly enriched for candidate genes associated with GL.

The annotation of 64 significant variants revealed the following classifications: 23.91% were intron variants, 23.37% were intergenic variants, 10.33% were downstream gene variants, 39.13% were upstream gene variants, and 3.26% were synonymous variants. Furthermore, 17 candidate genes were identified within a ±500 Kb region surrounding the significant SNPs. These genes were analyzed using the GO and KEGG databases. The detailed information of candidate genes is presented in Table 3. The candidate genes were significantly enriched in 35 GO terms and 28 KEGG pathways, as illustrated in Figure 5A,B. Among these, the top ten significantly enriched KEGG pathways and GO terms were predominantly associated with hormone secretion, energy metabolism, and signal transduction. Key pathways included “ssc04912, GnRH signaling pathway”, “ssc04727, GABAergic synapse”, “ssc04913, Ovarian steroidogenesis”, and “ssc04914, Progesterone-mediated oocyte maturation”; Significant GO terms included “GO:0032154, cleavage furrow” and “GO:0006171, cAMP biosynthetic process”. Based on the enrichment analysis and literature review, *TROAP, RFX4,* and *ADCY6* emerged as the most compelling candidate genes. Among these, *ADCY6* was notably enriched in critical pathways such as GnRH signaling, GABAergic synapse, and ovarian steroidogenesis, which are vital for maintaining pregnancy and reproduction (Table 4).

## 4. Discussion

Gestation length (GL) is related to the litter traits of sows, such as total number born (TNB), number born alive (NBA), etc. Genetic factors contribute to gestation length [29,30], making it critical to unravel its genetic mechanism and clarify its relationship with litter traits for practical breeding programs. In this study, we analyzed the farrowing records of 7013 sows’ first parity litter traits and genotyped 3005 sows of interest with complete phenotypes and pedigrees. Subsequently, we evaluated the impact of GL on litter traits and conducted imputed whole genome sequence-based GWAS to fine-map QTLs and identify candidate genes.

Phenotypic analysis revealed that sows with GL ≤ 114 days accounted for 49.1%, while 50.9% had GL > 114 days in this study cohort. The results showed that the phenotypic correlations between GL and litter traits were generally low, except the correlation between gestational length (GL) and stillbirths (*cor* = −0.201, *p*-value = 5.56 × 10^−91^). This may be attributed to the GL concentrated between GL 114 and 116 days, which caused a bias in the result. Even so, we discovered that extending the gestation period from 109 to 114 days led to an increased litter size, while GL > 114 days decreased the litter size (Figure S1). These findings align with prior studies [29,31], suggesting both excessively short and excessively long GL negatively impact the reproductive performance of sows.

The heritability of GL was estimated using a linear mixed model based on imputed whole genome sequence and complete pedigree data. The estimated heritability was 0.26 (genomic-based) and 0.22 (pedigree-based). The discrepancy between genomic-based heritability and pedigree-based heritability may be attributed to the varying degrees of pairwise relationships among individuals. Our results are consistent with previous studies in multi-breeds [32,33,34], confirming that GL is heritable and is controlled by some potential genes that need to be identified and mapped.

GWAS have become increasingly prominent in pig genetics research [35]. According to the Pig QTL database, 286 QTLs were recorded for GL in this study to enhance the detection capabilities and identify key mutations influencing GL by imputing a 50 K genotypic panel to whole-genome sequences. This imputation utilized a high-quality and reliable haplotype panel constructed by Tong et al. as a reference [19]. Following imputation, a GWAS based on the imputed whole-genome sequence was conducted, leading to the identification of 64 significant SNPs located on SSC5, SSC6, and SSC9. Seventeen candidate genes were annotated within ±0.5 Mb of these significant SNPs, with sixteen genes located on SSC5 and one on SSC9 (Table 3). Using the 2-LOD method, a large genomic region on SSC5 (from 12.8 Mb to 17.7 Mb) was identified. Additionally, two independent QTLs were fine-mapped within this region based on linkage disequilibrium analysis (QTL-1: 15290495–15392868 bp and QTL-2: 12868808–12949729 bp). We compared our findings with the pig QTL database and the QTLs reported by Hidalgo AM et al. and Onteru et al. [36,37], the significant genomic region (from 12.8 Mb to 17.7 Mb) on SSC5 for French Large White sows that had not been previously reported. The analysis suggests that the QTLs and candidate genes identified in our study are novel for GL in large white sows.

The genes involved in the reproductive process are of primary interest in studies of gestation length. Therefore, based on gene enrichment analysis and literature review, three compelling candidate genes were identified for GL, including *TROAP* (Trophinin Associated Protein), *RFX4* (Regulatory Factor X4), and *ADCY6* (Adenylate Cyclase 6).

*TROAP* (Trophinin Associated Protein): among these significant SNPs, seven significant SNPs were intron variants within the *TROAP* gene. GO analysis revealed that this gene is associated with protein binding and cell adhesion. The *TROAP* gene encodes a protein essential for cell proliferation, spindle assembly during mitosis, and centrosome integrity [38], and it has been reported to function in conjunction with bystin and trophinin within a cell adhesion molecule complex that mediates the initial attachment of the blastocyst to uterine epithelial cells during the embryo implantation, which is critical for human embryonic development [39]. These findings suggest that the *TROAP* gene plays a crucial role in embryonic growth and development and should be regarded as a potential candidate gene for GL.

*RFX4* (Regulatory Factor X4): one identified SNP (5_13748239) was an intron variant of the *RFX4* gene. This gene is implicated in neural tube ciliogenesis during embryogenesis and plays an important role in early brain development [40]. Previous GWAS on indigenous Chinese cattle and Brangus heifers also identified *RFX4* as a gene influencing fertility [41,42,43]. These studies suggest that RFX4 may impact gestation length by affecting embryonic development.

*ADCY6* (Adenylate Cyclase 6) gene is a member of the membrane-bound adenylate cyclase (AC) family, which catalyzes the conversion of adenosine triphosphate (ATP) to cyclic adenosine monophosphate (cAMP), a critical molecule for estrogen secretion stimulated by follicle-stimulating hormone (FSH) and luteinizing hormone (LH) [44,45]. According to the enrichment analysis, *ADCY6* is significantly involved in 12 KEGG pathways and 7 GO terms, including ovarian steroidogenesis, progesterone-mediated oocyte maturation, the GnRH signaling pathway, adenylate cyclase activity, and the cAMP biosynthetic process. Ovarian steroidogenesis begins with the conversion of cholesterol to pregnenolone, leading to the production of progesterone, a hormone essential for maintaining pregnancy and preventing spontaneous preterm birth (SPB) [46]. The GnRH signaling pathway plays a crucial role in regulating gonadotropins. Gonadotropin-releasing hormone (GnRH) is a pivotal regulator of the two primary gonadotropins, follicle-stimulating hormone (FSH) and luteinizing hormone (LH), which are critical for estrogen synthesis, including estradiol and progesterone. Moreover, *ADCY6* gene expression is linked to oocyte maturation [47]. The gene is targeted by miR-96-5p, which may regulate oxytocin and protein binding through *ADCY6* [48]. These findings indicate that *ADCY6* plays a pivotal role in regulating reproductive processes, including oocyte maturation, gestation, and parturition. However, further experimental validation is needed to confirm its function.

Overall, our results identified three compelling candidate genes, including *TROAP*, *RFX4*, and *ADCY6*, as well as two novel QTLs with gestation length traits in French Large White pigs. However, the study faced certain limitations, including the characteristics of the discovery data set and the incomplete understanding of the functional roles these genes play in regulating gestation length. Further studies are required to confirm the findings.

## 5. Conclusions

In summary, gestation length is a heritable and essential trait that plays a pivotal role in the reproductive performance of sows, with heritability estimates from 0.22 to 0.26. Through GWAS, we identified 64 significant SNPs associated with gestation length alongside two novel QTLs and three promising genes: *TROAP, RFX4,* and *ADCY6*. While this study has certain limitations, the findings offer valuable insights into the genetic mechanisms underlying gestation length and establish a solid foundation for future research and breeding strategies aimed at optimizing this critical trait.

## Figures and Tables

**Table 1:** Correlation analysis between gestation length and reproductive traits in Large White pigs.

Traits	GL Category	
	≤114 Days	≥114 Days
TNB	−0.01 ^ns^	−0.116 *
NBA	0.114 *	−0.127 *
NHB	0.128 *	−0.099 *
SN	−0.201 *	0.042 *
ABW (kg)	0.014 ^ns^	0.090 *
LWB (kg)	0.111 *	−0.076 *

TNB: total number born; NBA: number born alive; NHB: number of healthy piglets born; SN: stillborn number; ABW: average birth weight; LWB: litter weight at birth; GL: gestation length. *: significant. ns: not significant.

**Table 2:** Significant SNPs associated with gestation length traits identified through GWAS.

SSC	N	M	Position	Top SNP	
				SNP	*p*-Value
5	61	2	12861377–17510975	5_15295033	4.69 × 10^−8^
				5_12923575	4.92 × 10^−8^
6	1	0	34420154	6_34420154	2.73 × 10^−7^
9	2	0	48574739–48574749	9_48574739	8.34 × 10^−7^

SSC: The position of SNP on *Sus scrofa* chromosome; N: number of significant SNPs (*p* < 1 × 10^−6^); M: number of significant SNPs (*p* < 5 × 10^−8^); Position: Range of significant SNP in Ensembl; Top SNP: The most significant SNP within the significant regions.

**Table 3:** Details of candidate genes identified in the ±500 Kb regions around significant SNPs.

SSC	Candidate Genes	Position (bp)	N	Consequence
5	ABTB3	12861377–12928271	4	Intron variant
	*ADCY6*	14837925	1	Intron variant
	*BTBD11*	12861377–12928271	4	Intron variant
	*C1QL4*	15303213–15303558	3	Downstream gene variant
	*DNAJC22*	15319182	1	Upstream gene variant
	*FAM186B*	15540506–15540529	2	Downstream gene variant
	*FIGNL2*	17198330–17211201	6	Intron variant; Upstream gene variant
	*KCNH3*	15491006	1	Upstream gene variant
	*KRT80*	17479435–17510975	5	Downstream gene variant; Upstream gene variant
	*LIMA1*	16126979–16133781	2	Intron variant
	*LOC110260652*	13761753	1	Upstream gene variant;
	*LOC110260837*	13761753	1	Downstream gene variant
	*PRPH*	15293456–15293521	3	Downstream gene variant
	*RFX4*	13748239–13761753	2	Intron variant Upstream gene variant
	*SPMIP11*	14837925	1	Downstream gene variant
	*TROAP*	15296715–15303558	27	Upstream gene variant; Synonymous variant; Intron variant; Missense variant; Stop lost
9	*SORL1*	48574739–48574749	2	Intron variant

SSC: The position of SNP on *Sus scrofa* chromosome; N: number of SNPs within the gene.

**Table 4:** The significant KEGG pathways and GO terms.

Term	Database	ID	*p*-Value	Genes
Vasopressin-regulated water reabsorption	KEGG	ssc04962	2.12 × 10^−2^	*ADCY6*
Endocrine and other factor-regulated calcium reabsorption	KEGG	ssc04961	2.26 × 10^−2^	*ADCY6*
Ovarian steroidogenesis	KEGG	ssc04913	2.31 × 10^−2^	*ADCY6*
Regulation of lipolysis in adipocytes	KEGG	ssc04923	2.69 × 10^−2^	*ADCY6*
Cortisol synthesis and secretion	KEGG	ssc04927	3.02 × 10^−2^	*ADCY6*
Taste transduction	KEGG	ssc04742	3.25 × 10^−2^	*ADCY6*
Renin secretion	KEGG	ssc04924	3.35 × 10^−2^	*ADCY6*
Bile secretion	KEGG	ssc04976	3.35 × 10^−2^	*ADCY6*
GnRH signaling pathway	KEGG	ssc04912	3.94 × 10^−2^	*ADCY6*
GABAergic synapse	KEGG	ssc04727	3.94 × 10^−2^	*ADCY6*
cellular response to forskolin	GO	GO:1904322	3.88 × 10^−3^	*ADCY6*
ruffle organization	GO	GO:0031529	3.88 × 10^−3^	*LIMA1*
adenylate cyclase activity	GO	GO:0004016	5.33 × 10^−3^	*ADCY6*
cAMP biosynthetic process	GO	GO:0006171	5.33 × 10^−3^	*ADCY6*
cellular response to prostaglandin E stimulus	GO	GO:0071380	5.33 × 10^−3^	*ADCY6*
dopamine receptor signaling pathway	GO	GO:0007212	5.81 × 10^−3^	*ADCY6*
negative regulation of fibroblast proliferation	GO	GO:0048147	5.81 × 10^−3^	*C1QL4*
regulation of blood vessel diameter	GO	GO:0097746	6.78 × 10^−3^	*ADCY6*
intermediate filament cytoskeleton organization	GO	GO:0045104	7.26 × 10^−3^	*PRPH*
cleavage furrow	GO	GO:0032154	1.06 × 10^−2^	*LIMA1*
cell adhesion	GO	GO:0007155	3.66 × 10^−2^	*TROAP*
telencephalon development	GO	GO:0021537	3.15× 10^−3^	*RFX4*

## Data Availability

The raw data has been uploaded to NCBI but is not yet publicly accessible; for further inquiries, please contact the corresponding authors.

## Supplementary Materials

The following supporting information can be downloaded at: https://www.mdpi.com/article/10.3390/ani15030447/s1, Figure S1: The relationship between gestation length and reproductive traits; Figure S2: Comparison of gestation length across genotypes of the two leading variants on SSC5; Figure S3: Principal component analysis (PCA) based on genomic data.
