# Supplementary material for: Genome-Wide Association Study Pinpoints Novel Candidate Genes Associated with the Gestation Length of the First Parity in French Large White Sows
Source: Animals (Basel). 2025 Feb 6;15(3):447. doi: 10.3390/ani15030447 (PMC11815982; doi:10.3390/ani15030447)
Supplement: Supplementary file 1 [file animals-15-00447-s001.zip › animals-3328193-supplementary.pdf]

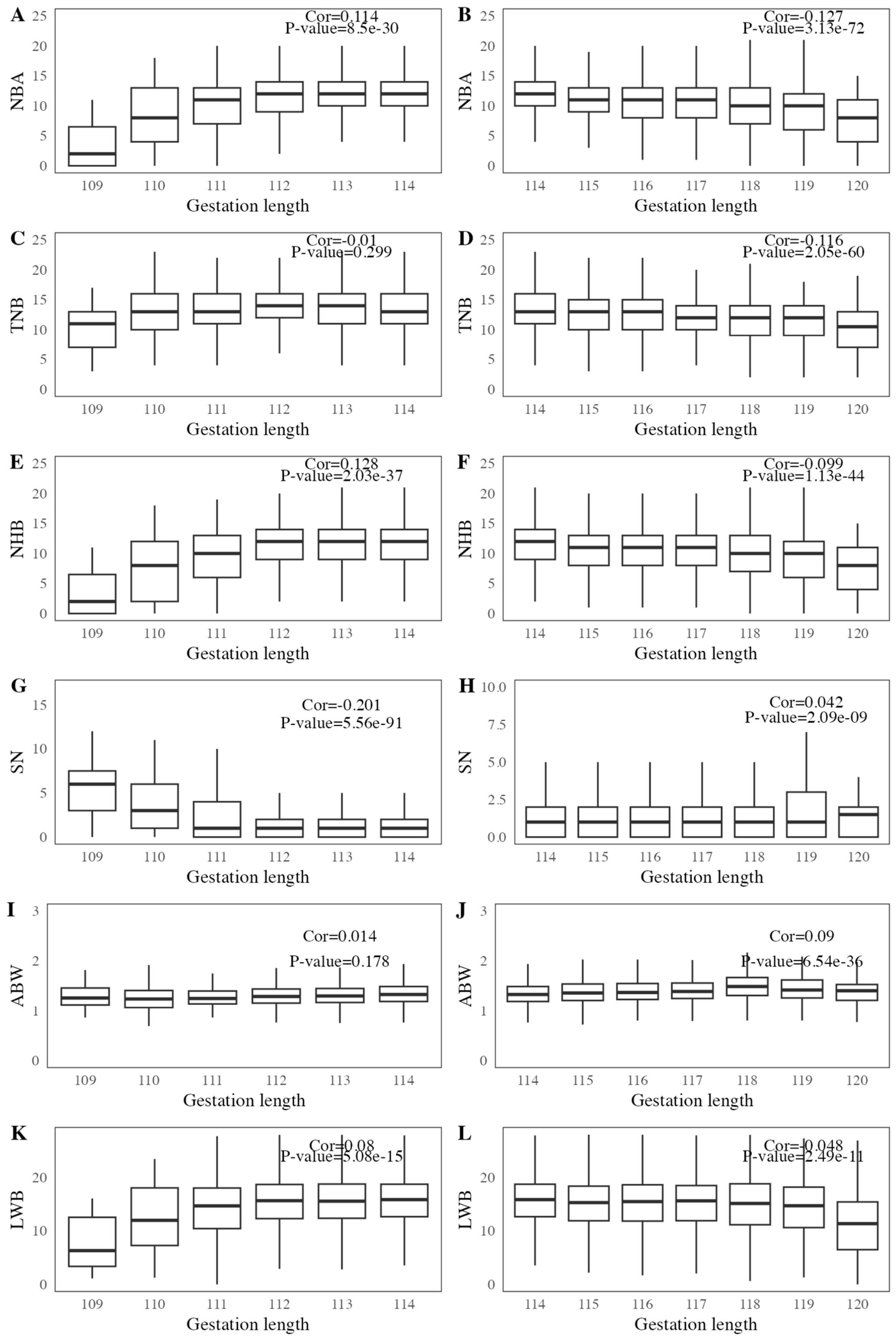

Figure S1. The relationship between gestation length and reproductive traits. Panels A–L illustrate the relationship between gestation length (GL) and various reproductive traits, including the number of piglets born alive, total number of piglets born, number of healthy piglets born, number of stillborn piglets, average birth weight, and litter weight at birth, respectively.

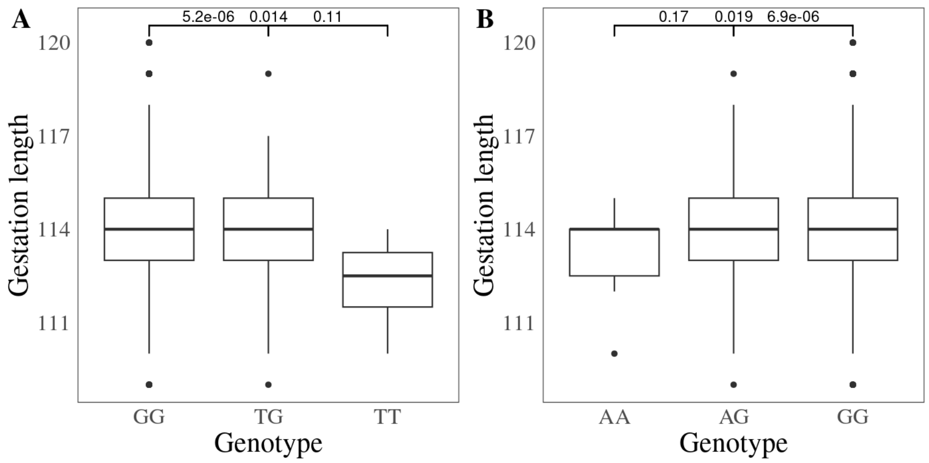

Figure S2. Comparison of gestation length across genotypes of the two leading variants on SSC5. Panel A illustrates the variation in gestation length among different genotypes of SNP chr5_15295033, while Panel B shows the corresponding differences for SNP chr5_12923575.

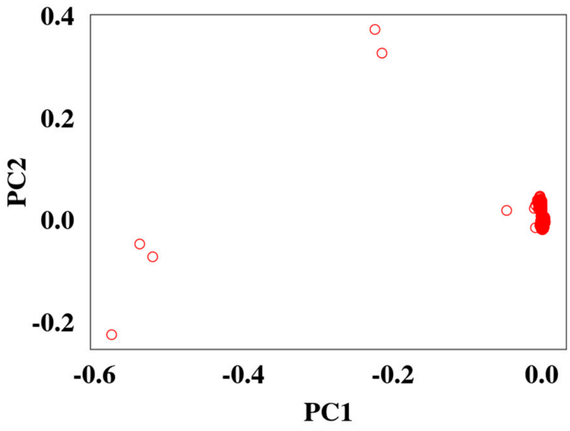

Figure S3. Principal component analysis (PCA) based on genomic data. PC1 represents the first principal component and PC2 represents the second principal component.
